# Programmed death ligand-1 and MET co-expression is a poor prognostic factor in gastric cancers after resection

**DOI:** 10.18632/oncotarget.19390

**Published:** 2017-07-19

**Authors:** Mi Jung Kwon, Kab-Choong Kim, Eun Sook Nam, Seong Jin Cho, Hye-Rim Park, Soo Kee Min, Jinwon Seo, Ji-Young Choe, Hye Kyung Lee, Ho Suk Kang, Kyueng-Whan Min

**Affiliations:** ^1^ Department of Pathology, Hallym University Sacred Heart Hospital, Hallym University College of Medicine, Gyeonggi-do 431-796, Republic of Korea; ^2^ Department of Surgery, Hallym University Sacred Heart Hospital, Hallym University College of Medicine, Gyeonggi-do 431-796, Republic of Korea; ^3^ Department of Pathology, Kangdong Sacred Heart Hospital, Hallym University College of Medicine, Seoul 134-701, Republic of Korea; ^4^ Department of Internal Medicine, Hallym University Sacred Heart Hospital, Hallym University College of Medicine, Gyeonggi-do 431-796, Republic of Korea; ^5^ Department of Pathology, Hanyang University Guri Hospital, Hanyang University College of Medicine, Gyeonggi-do 11923, Republic of Korea

**Keywords:** gastric cancer, programmed death ligand-1 protein, MET protein, prognosis, microsatellite instability

## Abstract

Programmed death-ligand 1 (PD-L1) plays an essential protein for immune evasion, contributing to tumor development and progression. Recent studies have reported MET as an upregulator for PD-L1 overexpression through an oncogenic pathway. However, an association between PD-L1 expression with MET has not been reported in gastric cancer.The prognostic significance of PD-L1 and its association with Epstein-Barr virus (EBV), microsatellite instability (MSI), and mucin phenotype remain controversial.

We performed *in situ* hybridization for EBV-encoded RNA and immunohistochemistry in tissue microarrays for 394 gastric cancers. A multiplex polymerase chain reaction with five quasimonomorphic markers was performed for MSI.

PD-L1 expression was observed in 123 cases (31.2%), and clinicopathological features such as MET overexpression, high pT stage, and a lack of lymphatic invasion represent significant risk factors associated with PD-L1 overexpression in gastric cancers. No associations of EBV, MSI, or mucin phenotype with PD-L1 expression were statistically significant. PD-L1 expression was a strong indicator for worse overall survival (OS) but borderline significant in disease-free survival (DFS). A combined analysis of PD-L1 and MET expression indicated that the PD-L1+/MET+ subgroup showed the worst prognosis when compared to the PD-L1-/MET- subgroup, which had the best clinical outcome. Furthermore, PD-L1 overexpression exhibited poor prognosis in terms of both OS and DFS in EBV-negative, microsatellite stable, and intestinal mucin phenotype tumors. In conclusion, this is the first study to evaluate the overexpression of MET as a risk factor for PD-L1 positivity in gastric cancer tissue as well as the reliability and prognostic relevance of PD-L1/MET co-expression after surgery.

## INTRODUCTION

Gastric cancer is the fourth most common human malignancy and the second leading cause of global cancer mortality, and is notorious for a dismal prognosis with a five-year survival rate of less than 40% despite multimodality treatments [1]. In the Korean population, gastric cancer also represents the fourth most prevalent cause of cancer-related deaths, accounting for 14% of all cancers. Gastric cancer also has a high incidence, with 34,331 newly diagnosed cases in 2016 and 7,054 patients that succumbed to gastric cancers in Korea [2]. The limited number of treatment options and unsatisfactory clinical outcomes highlight the need for new reliable predictors of survival and novel therapeutic targets for gastric cancer.

Immune evasion by cancer cells plays an important role in the development and progression of tumors [3]. An immunogenic interaction exists between the host and the tumor, and the ability of the tumor to evade immune recognition can determine the clinical course of the disease [3]. Cancer cells can express many immune inhibitory signaling proteins to induce immune cell dysfunction and apoptosis. One of these inhibitory molecules is programmed death-ligand 1 (PD-L1; also known as B7-H1 or CD274) that is expressed on tumor cells, which then binds to programmed death 1 (PD-1) that is expressed on T-cells, B-cells, dendritic cells, and natural killer T-cells to suppress anti-cancer immunity and to enable neoplastic growth [3]. Increased PD-L1 expression and its interaction to PD-1 have been associated with poor prognosis in several cancers including gastric, esophageal, lung, and renal cell cancers [4-8]; however, its prognostic value is still controversial. Inhibition of PD-L1 with antibodies can improve the overall survival rate in patients with these cancers [9-11]. However, a proportion of PD-L1-negative patients also benefits from anti-PD-1 therapy. This suggests that PD-L1 expression per se has not been fully accounted for its survival benefit, and there may be other molecular determinants involved in PD-L1 expression and its therapeutic effects. Considering the clinical importance of PD-L1, there is great interest in understanding the mechanisms that regulate its expression [5]. The MET/hepatocyte growth factor (HGF) pathway is a potential candidate, since MET-induced signaling has been reported to induce PD-L1 expression in renal cell cancers [12]. Binding of HGF to MET induces the phosphorylation of the docking site and stimulates mitogen-activated protein kinase (MAPK) and phosphatidylinositol 3-kinase (PI3K)/Akt pathways [13], which are one of the drivers that upregulate PD-L1 expression in melanoma and non-small cell lung cancer [6, 14]. In addition, PD-L1 upregulation has been reported in Epstein-Barr virus (EBV)-associated malignancies, including gastric cancers, nasopharyngeal carcinoma, and malignant lymphoma [15-18]. Microsatellite instability-high (MSI-H) has also been shown to promote PD-L1 overexpression in colorectal and gastric cancers [15]. Furthermore, unclassified mucin phenotype has been reported to be associated with PD-L1 overexpression in gastric cancers [16]. Nevertheless, a correlation between PD-L1 expression with EBV, MSI, or mucin phenotype has not been fully evaluated in Korean patients with gastric cancer. In this study, we focused on the aberrant co-overexpression of PD-L1 and MET in gastric cancers to determine whether this aberrant expression was associated with the clinical outcome. Since gastric cancers are therapy-resistant and they have few established prognostic and predictive markers, studies on PD-L1 and its related oncogenic pathway in gastric cancers may provide insight into novel treatment modalities.

In the present study, we investigated the expression of PD-L1 and its prognostic relevance in gastric cancers, and explored the clinicopathological factors affecting PD-L1 overexpression. Further stratified survival analyses of PD-L1 expression with regards to MET expression, EBV, MSI status, and mucin phenotype were conducted.

## RESULTS

### Patient characteristics

The male-to-female ratio was 2.3:1. The mean age of the patients with gastric cancer was 60 years (median, 63 years; range, 23-89 years). The patient cohort consisted predominantly of those with middle to distal stomach tumors (93.9%). Tumors were either located at the upper third (n = 19, 4.8%), middle third (n = 187, 47.5%), lower third (n = 183, 46.4%), or the whole stomach (n = 5, 1.3%).Seventy-seven tumors (19.5%) were well-differentiated tubular adenocarcinoma, 94 (23.9%) were moderately differentiated tubular adenocarcinoma, 160 (40.6%) were poorly differentiated tubular adenocarcinoma, 56 (14.2%) were signet ring cell carcinoma, and 7 (1.8%) were mucinous adenocarcinoma. Intestinal, diffuse, and mixed type gastric adenocarcinomas according to the Lauren classification system comprised 51.5%, 32.0%, and 16.5%, respectively, of the samples. Of the 394 gastric cancers, 232 (58.9%) were diagnosed as stage I, 55 (14.0%) as stage II, 94 (23.8%) as stage III, and 13 (3.3%) as stage IV. Out of these patients, postoperative adjuvant chemotherapy was administered in 21 (9.1%) with stage I, 35 (63.6%) with stage II, 64 (68.1%) with stage III, and 9 (69.2%) with stage IV. Tumors consisted of pT1 (n = 209, 53.0%), pT2 (n = 40, 10.2%), pT3 (n = 98, 24.9%), and pT4 (n = 47, 11.9%). The pN status were pN0 (n = 241, 61.2%), pN1 (n = 45, 11.4%), pN2 (n = 44, 11.2%), and pN3 (n = 64, 16.2%). The mean follow-up period was 94.1 ± 34.4 months. A total of 128 (32.5%) patients experienced a recurrence, of which 117 patients (91.4%) died and 11 patients (8.6%) are alive. One hundred twenty-five patients (31.7%) patients died of the disease, of which 8 patients (6.4%) did not have any recurrence.

### PD-L1 expression and its correlation with clinicopathological features

**Figure 1:**
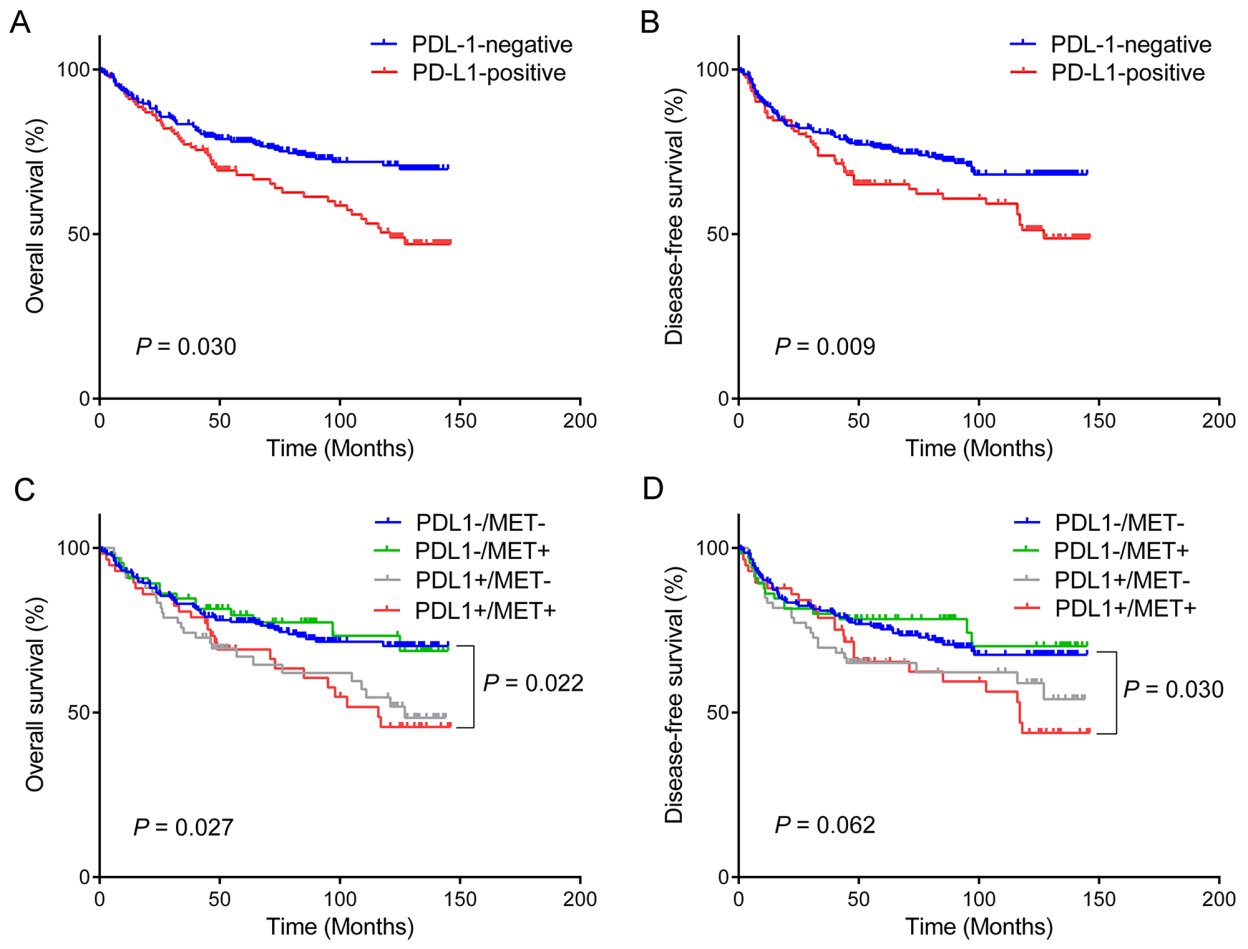
Kaplan–Meier survival curves according to PD-L1 expression status for overall survival **(A)** and disease-free survival **(B)**. Combined analyses of PD-L1/MET expression demonstrate that patients with PD-L1-positive/MET-positive (PD-L1+/MET+) gastric cancer have the worst overall survival **(C)** and disease-free **(D)** survival, whereas those with PD-L1-negative/MET-negative (PD-L1-/MET-) tumor have the most favorable prognosis.

The clinical and pathological characteristics of the patients were analyzed according to their PD-L1 and MET status (Table 1). Tumor tissue samples from 123 patients (31.2%) were PD-L1-positive, and the remaining specimens (271 patients, 68.8%) were PD-L1-negative. While MET was positive in 122 cases (31.0%), 57 (46.7%) of which were PD-L1-positive, HGF was positive in 125 gastric cancer (31.7%) samples (Figure 1I-1L), 49 (39.2%) of which were PD-L1-positive. PD-L1 positivity was well correlated with both MET expression and HGF expression (*P* < 0.001 and *P* = 0.020, respectively). EBV was positive in 26 of 394 samples (6.6%), and of these EBV-positive cases, 11 (42.3%) were PD-L1-positive. MSI-H was observed in 37 (9.4%) of the 394 gastric cancers, whereas microsatellite-stable (MSS) status was observed in 357 cases (90.6%). Of the 37 MSI-H cases, 9 (24.3%) were PD-L1-positive. There were no correlations between PD-L1 positivity and EBV or MSI-H (*P* = 0.207 and *P* = 0.456, respectively).

**Table 1:** Correlations of PD-L1 with clinicopathological characteristics

Variable	Total	PD-L1 expression		*P*
		Positive	Negative	
	N = 394 (%)	n = 123 (31.2%)	n = 271 (68.8%)	
Gender				0.813
Male	274 (69.5)	87 (70.7)	187 (69.0)	
Female	120 (30.5)	36 (29.3)	84 (31.0)	
Age (y)				0.943
<60	158 (40.1)	49 (39.8)	109 (40.2)	
≥60	236 (59.9)	74 (60.2)	162 (59.8)	
Tumor location				0.294
Upper third	19 (4.8)	8 (6.5)	11 (4.1)	
Middle third	187 (47.5)	60 (48.8)	127 (46.9)	
Lower third	183 (46.4)	52 (42.3)	131 (48.3)	
Whole stomach	5 (1.3)	3 (2.4)	2 (0.7)	
Tumor size (cm)				0.743
<5	264 (67.0)	81 (65.9)	183 (67.5)	
≥5	130 (33.0)	42 (34.1)	88 (32.5)	
Lauren classification				0.623
Intestinal	203 (51.5)	64 (52.0)	139 (51.3)	
Diffuse	126 (32.0)	36 (29.3)	90 (33.2)	
Mixed	65 (16.5)	23 (18.7)	42 (15.5)	
Histology				0.575
Differentiated	168 (42.6)	55 (44.7)	113 (41.7)	
Undifferentiated	226 (57.4)	68 (55.3)	158 (58.3)	
Mucin phenotype				0.067
Intestinal	157 (39.8)	56 (45.5)	101 (37.3)	
Gastric	57 (14.5)	12 (9.8)	84 (31.0)	
Mixed	124 (31.5)	40 (32.5)	45 (16.6)	
Unclassified	56 (14.2)	15 (12.2)	41 (15.1)	
pT-category				0.286
T1-2	249 (63.2)	73 (59.3)	176 (64.9)	
T3-4	145 (36.8)	50 (40.7)	95 (35.1)	
pN-category				0.738
N0	241 (61.2)	77 (62.6)	164 (60.5)	
N1-3	153 (38.8)	46 (37.4)	107 (39.5)	
AJCC stage				1.000
I-II	287 (72.8)	90 (73.2)	197 (72.7)	
III-IV	107 (27.2)	33 (26.8)	74 (27.3)	
Lymphatic invasion				0.043
Present	144 (36.5)	36 (29.3)	108 (39.9)	
Absent	250 (63.5)	87 (70.7)	163 (60.1)	
Vascular invasion				0.296
Present	43 (10.9)	10 (8.1)	33 (12.2)	
Absent	351 (89.1)	113 (91.9)	238 (87.8)	
Perineural invasion				0.883
Present	63 (16.0)	19 (15.4)	44 (16.2)	
Absent	331 (84.0)	104 (84.6)	227 (83.8)	
EBV status				0.207
Positive	26 (6.6)	11 (8.9)	15 (5.5)	
Negative	368 (93.4)	112 (91.1)	256 (94.5)	
MSI status				0.456
MSS	357 (90.6)	114 (92.7)	243 (89.7)	
MSI-H	37 (9.4)	9 (7.3)	28 (10.3)	
MET				<0.001
Positive	122 (31.0)	57 (46.3)	65 (24.0)	
Negative	272 (69.0)	66 (53.7)	206 (76.0)	
HGF				0.020
Positive	125 (31.7)	49 (39.8)	76 (28.0)	
Negative	76 (19.3)	74 (60.2)	195 (72.0)	

EBV, Epstein-Barr virus; MSI, microsatellite instability.

The expression levels of CDX-2, MUC2, MUC5AC, and MUC6 were demonstrated in 280 (71.1%), 70 (82.2%), 181 (45.9%), and 63 (16.0%) of 394 cases, respectively. Among these markers, PD-L1 positivity was most frequently co-expressed with CDX-2 (78.0%, 96/123), followed by MUC5AC (42.3%), MUC6 (20.0%), and MUC2 (18.7%). The tendency for PD-L1 to be co-expressed with CDX-2 was statistically significant (*P* = 0.042), whereas there were no associations with other mucin markers (data not shown). Taking into account of the observed combinations of these phenotypic markers, 394 gastric cancers were classified into 157 intestinal phenotypes (39.8%), 57 gastric phenotypes (14.5%), 124 mixed phenotypes (31.5%), and 56 unclassified mucin phenotypes (14.2%), where PD-L1 positivity was most commonly observed in 56 intestinal phenotypes (45.5%), followed by 40 mixed phenotypes (32.5%), 15 unclassified phenotypes (12.2%), and 12 gastric phenotypes (9.8%). This difference of PD-L1 expression among the mucin phenotype was marginally statistically significant (*P* = 0.067).

PD-L1 positivity was not significantly correlated with any clinical characteristics except for lymphatic invasion. However, PD-L1 positivity was inversely correlated with lymphatic invasion (*P* = 0.043).

The clinicopathological factors affecting PD-L1 overexpression were investigated by multivariate analyses using a logistic regression model (Table 2). The multivariate analyses revealed that high pT stage, a lack of lymphatic invasion, and MET overexpression were the independent predictive clinicopathological factors for PD-L1 overexpression (*P* = 0.041, odds ratio = 2.208, confidence interval (95% CI) = 1.032–4.723; *P* = 0.032, odds ratio = 0.502, 95% CI = 0.268–0.941; and *P* < 0.001, odds ratio = 2.688, 95% CI = 1.641–4.403; respectively).

**Table 2:** Clinicopathological factors affecting PD-L1 overexpression by multivariate analysis

	PD-L1 expression		*P* value
	Odds ratio	95% CI	
Gender (Male vs. Female)	1.066	0.647–1.757	0.803
Age (y) (<60 vs. ≥60)	0.913	0.565–1.475	0.709
Tumor location (Proximal vs. Distal)	0.603	0.329–1.106	0.102
Tumor size (cm) (<5 vs. ≥5)	1.041	0.575–1.885	0.893
Histology (Differentiated vs. Undifferentiated)	0.853	0.520–1.399	0.529
Mucin phenotype (Other vs. Unclassified)	0.883	0.427–1.828	0.737
pT stage (T1-2 vs. T3-4)	2.208	1.032–4.723	0.041
pN stage (N0 vs. N1-3)	0.950	0.417–2.166	0.903
Stage (I,II vs. III, IV)	0.798	0.317–2.006	0.631
Lymphatic invasion (Absent vs. Present)	0.502	0.268–0.941	0.032
Vascular invasion (Absent vs. Present)	0.759	0.313–1.839	0.541
Perineural invasion (Absent vs. Present)	1.332	0.631–2.814	0.452
EBV (Negative vs. Positive)	1.615	0.669–3.899	0.286
MSI status (MSS vs. MSI-H)	0.655	0.271–1.587	0.349
MET (Negative vs. Positive)	2.688	1.641–4.403	<0.001
HGF (Negative vs. Positive)	1.547	0.941–2.541	0.085

EBV, Epstein-Barr virus; MSI, microsatellite instability.

### Prognostic implications of PD-L1 expression

The overexpression of PD-L1 had a prognostic impact on the overall survival (OS) and disease-free survival (DFS) rate in patients with gastric cancers (Table 3). Patients with PD-L1 overexpression had shorter OS and DFS (mean of 97.9 and 96.6 months, respectively) than those with PD-L1 negativity (mean of 113.5 and 110.1 months, respectively) (*P* = 0.003 and *P =* 0.009, respectively) (Figure 1A-1B). Clinical and pathological variables such as gender (*P* = 0.023), age (*P* = 0.004), tumor size (*P* < 0.001), unclassified mucin phenotype (*P* = 0.006), advanced stage (*P* < 0.001), lymphatic invasion (*P* < 0.001), vascular invasion (*P* = 0.003), perineural invasion (*P* < 0.001), and MSI-H (*P* = 0.033) significantly affected OS. On the other hand, age (*P* = 0.046), tumor size (*P* < 0.001), unclassified mucin phenotype (*P* = 0.024), advanced stage (*P* < 0.001), lymphatic invasion (*P* < 0.001), vascular invasion (*P* = 0.003), perineural invasion (*P* < 0.001), and MSI-H (*P* = 0.022) were associated with DFS. Those clinical and pathological statistically proven variables were consistent with the previously reported prognostic factors in gastric cancers [19-21]. However, EBV positivity did not influence OS or DFS (*P* = 0.688 and *P* = 0.467, respectively).

**Table 3:** Overall survival and disease-free survival of patients with gastric carcinomas by univariate and multivariate analyses

	Overall survival				Disease-free survival			
	Univariate		Multivariate		Univariate		Multivariate	
	HR (95% CI)	*P*	HR 95% CI	*P*	HR 95% CI	*P*	H (95% CI)	*P*
Gender	0.617	0.023	0.697	0.101	0.706	0.087	0.764	0.200
Male vs. Female	(0.405–0.939)		(0.452–1.074)		(0.473–1.054)		(0.506–1.153)	
Age (y)	1.754	0.004	1.786	0.005	1.451	0.046	1.479	0.051
<60 vs. ≥60	(1.197–2.571)		(1.189–2.683)		(1.004–2.096)		(0.998–2.192)	
Tumor size (cm)	3.185	<0.001	1.853	0.006	0.692	<0.001	1.968	0.002
<5 vs. ≥5	(2.237–4.534)		(1.195–2.875)		(0.450–1.066)		(1.287–3.008)	
Histology	1.056	0.766	0.939	0.751	1.250	0.221	1.145	0.495
Differentiated vs. undifferentiated	(0.739–1.509)		(0.637–1.385)		(0.873–1.789)		(0.776–1.687)	
Mucin phenotype	1.823	0.006	1.254	0.364	1.644	0.024	1.136	0.612
Other vs. Unclassified	(1.183–2.810)		(0.769–2.045)		(1.061–2.546)		(0.694–1.859)	
Stage	4.324	<0.001	2.632	<0.001	4.372	<0.001	2.559	<0.001
I,II vs. III,IV	(3.038–6.153)		(1.627–4.258)		(3.084–6.197)		(1.602–4.088)	
Lymphatic invasion	2.148	<0.001	1.247	0.344	2.146	<0.001	1.138	0.573
Absent vs. Present	(1.503–3.070)		(0.790–1.969)		(1.510–3.048)		(0.726–1.786)	
Vascular invasion	2.050	0.003	0.852	0.582	2.003	0.003	0.955	0.866
Absent vs. Present	(1.267–3.314)		(0.483–1.505)		(1.253–3.202)		(0.558–1.634)	
Perineural invasion	2.599	<0.001	1.448	0.141	2.753	<0.001	1.513	0.085
Absent vs. Present	(1.732–3.899)		(0.885–2.369)		(1.861–4.072)		(0.944–2.426)	
PD-L1	1.716	0.003	1.709	0.006	1.598	0.009	1.437	0.058
Negative vs. Positive	(1.202–2.449)		(1.169–2.499)		(1.122–2.276)		(0.987–2.093)	
EBV	1.149	0.688	0.814	0.587	1.270	0.467	1.150	0.692
Negative vs. Positive	(0.583–2.265)		(0.388–1.709)		(0.666–2.423)		(0.576–2.293)	
MSI status	1.771	0.033	1.047	0.877	1.821	0.022	1.230	0.477
MSS vs. MSI-H	(1.046–2.998)		(0.583–1.879)		(1.091–3.041)		(0.695–2.176)	
MET	1.115	0.567	1.248	0.291	1.105	0.597	1.326	0.183
Negative vs. Positive	(0.767–1.620)		(0.827–1.885)		(0.764–1.598)		(0.876–2.008)	
HGF	1.100	0.616	0.991	0.964	1.076	0.698	0.863	0.479
Negative vs. Positive	(0.757–1.598)		(0.657–1.494)		(0.744–1.556)		(0.573–1.298)	

HR, hazard ratio; CI, confidence interval; EBV, Epstein-Barr virus; MSI, microsatellite instability.

By multivariate analysis, PD-L1 overexpression was confirmed to be an independent negative prognostic factor affecting OS (*P* = 0.006, hazard ratio (HR) = 1.709, 95% CI = 1.169–2.499), while there was borderline statistical significance with DFS (*P* = 0.058, HR = 1.437, 95% CI = 0.987–2.093). In addition, older age, larger tumor size, and advanced stage were also found to be independent prognostic factors for worse OS, whereas large tumor size and advanced stage were independent prognostic factors associated with DFS in gastric cancers. However, EBV status, MSI status, and MET or HGF expression were not independent prognostic factors for OS or DFS.

### Subgroup analysis of survival difference according to PD-L1 and MET expression

Since MET positivity was determined to be an independent predictive factor for PD-L1 overexpression, we subdivided PD-L1 and MET expression status into four subgroups: PDL1-/MET- (n = 206, 52.3%); PDL1-/MET+ (n = 65, 16.5%); PDL1+/MET- (n = 66, 16.7%); and PDL1+/MET+ (n = 57, 14.5%). A subgroup survival analysis according to the PD-L1 and MET expression status showed that co-expression of PD-L1 and MET was a significant prognostic factor of OS and DFS. Among the four subgroups, patients in the PDL1+/MET+ subgroup had a drastically worse prognosis in terms of both OS and DFS than those in the PDL1-/MET- subgroup. Patients in the PDL1+/MET+ subgroup showed shorter OS (mean = 97.2 months; 95% CI = 82.6–111.8) than those in the PDL1-/MET- subgroup (mean = 113.0 months; 95% CI = 105.7–120.2; *P* = 0.022), whereas the differences were not statistically significant when compared with the PDL1-/MET+ (mean = 115.4 months; 95% CI = 102.6–128.1; *P* = 0.052) and PDL1+/MET- subgroups (mean = 97.5 months; 95% CI 83.9–111.2; *P* = 0.849) (Figure 1C). Similarly, patients in the PDL1+/MET+ subgroup showed shorter DFS (mean = 95.6 months; 95% CI = 80.7–110.5) than those in the PDL1-/MET- subgroup (mean = 109.4 months; 95% CI 101.7–117.0; *P* = 0.030), while the differences were not statistically significant compared with those in the PDL1-/MET+ (mean = 112.5 months; 95% CI = 98.7–126.3; *P* = 0.053) and PDL1+/MET- subgroups (mean = 96.9 months; 95% CI = 82.4–111.3; *P* = 0.698) (Figure 1D).

The PD-L1/MET status and the abovementioned variables (gender, age, tumor size, mucin phenotype, stage, lymphatic invasion, vascular invasion, perineural invasion, or MSI status) that correlated significantly with OS or DFS on the univariate analyses were further analyzed by multivariate analyses. In the multivariate analyses, PDL1+/MET+ overexpression was a worse independent prognostic factor for OS (HR = 1.288, 95% CI = 1.104–1.502, *P* = 0.001) and DFS (HR = 1.214, 95% CI = 1.042–1.413, *P* = 0.013).

### Prognostic impact of PD-L1 expression according to EBV, MSI, and mucin phenotypes

**Figure 2:**
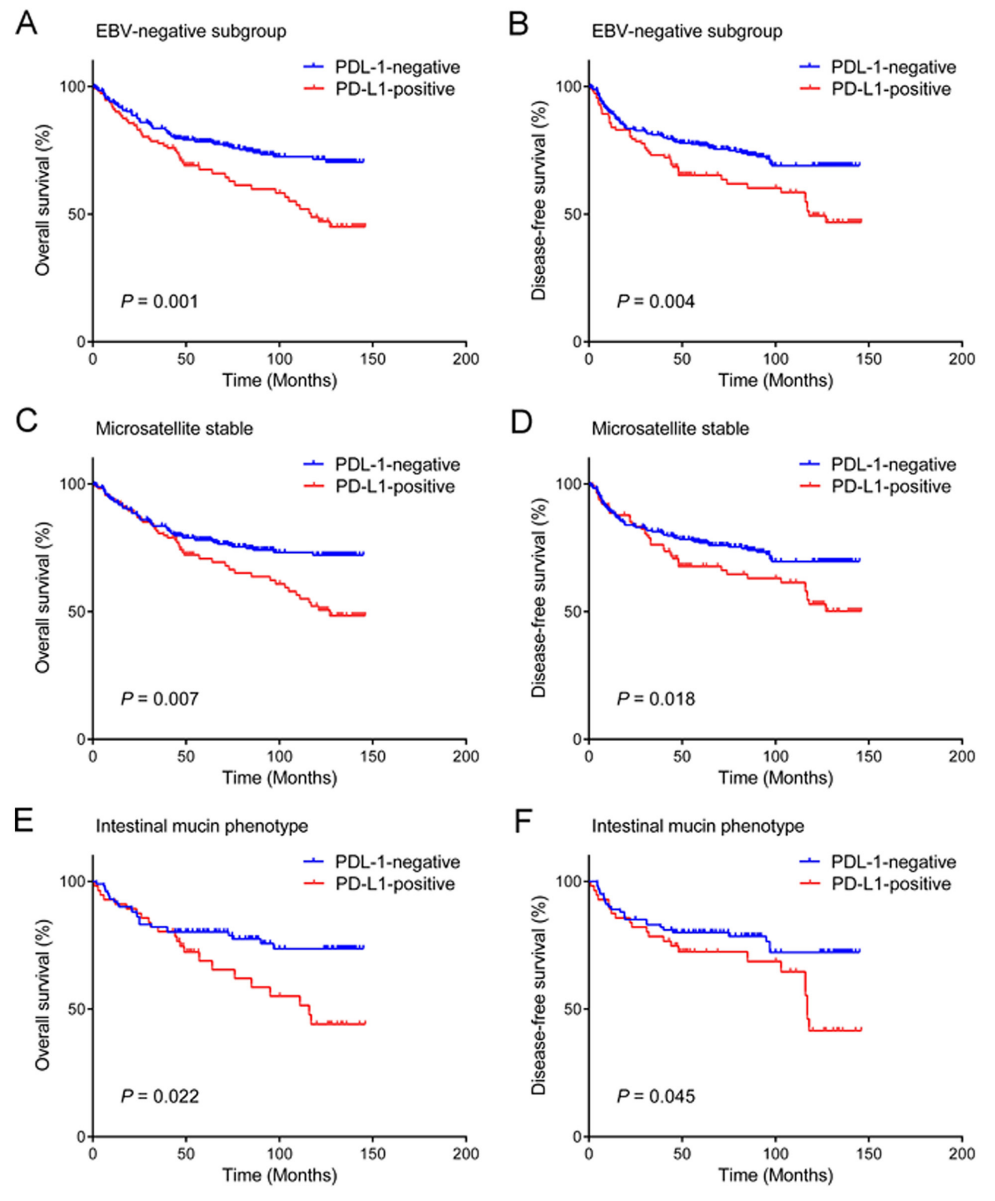
Prognostic impact of PD-L1 expression in gastric cancer according to EBV, MSI status, and mucin phenotype PD-L1 expression predicts unfavorable overall survival and disease-free survival of patients with EBV negativity **(A, B)**, microsatellite stable tumors **(C), (D)**, and intestinal mucin phenotype **(E, F).**

We further analyzed the prognostic value of PD-L1 expression for OS and DFS according to EBV, MSI status, or mucin phenotype (Figure 2). In EBV-negative gastric cancers, the OS and DFS were significantly worse in patients with PD-L1-positive tumors compared to those with PD-L1-negative tumors (*P* = 0.001 and *P* = 0.004, respectively; 96.4 ± 5.4 months vs. 114.2 ± 3.3 months for OS; and 95.4 ± 5.7 months vs. 111.1 ± 3.5 months for DFS). However, there were no significant differences in the OS or DFS for EBV-positive gastric cancers (*P* = 0.694 and *P* = 0.743, respectively).

For MSS gastric cancers, the OS and DFS were significantly worse in patients with PD-L1-positive tumors compared to those with PD-L1-negative tumors (*P* = 0.007 and *P* = 0.018, respectively; 101.1 ± 5.2 months vs. 114.7 ± 3.4 months for OS; 99.8± 5.4 months vs. 111.7 ± 3.6 months for DFS). There was also significant difference in the DFS in MSI-H gastric cancers (*P* = 0.041), while there was borderline statistical significance with OS (*P* = 0.057).

Furthermore, for the mucin phenotypes, PD-L1 positivity was correlated with shorter OS or DFS in the patients with gastric cancer showing the intestinal mucin phenotype (*P* = 0.022 and *P* = 0.045, respectively). In those with the mixed mucin type, PD-L1 positivity was associated only with decreased DFS (*P* = 0.045) but not with OS (*P* = 0.100). PD-L1 expression was not statistically associated with OS or DFS in other mucin phenotypes such as gastric type (*P* = 0.268 and *P* = 0.428) and unclassified type (*P* = 0.500 and *P* = 0.866).

## DISCUSSION

This study demonstrated that PD-L1 expression, comprising one third of gastric cancers, could be a strong prognostic indicator in patients with gastric cancer. Clinicopathological features such as MET overexpression, high pT stage, and a lack of lymphatic invasion were revealed to be significant risk factors that can be used to predict PD-L1 overexpression in gastric cancers. A combined analysis of PD-L1 and MET expressions indicated that the PD-L1+/MET+ subgroup showed the worst prognosis when compared to the PD-L1-/MET- subgroup, which showed the best clinical outcome. To the best of our knowledge, this is the first study to evaluate the overexpression of MET as a risk factor for the presence of PD-L1 positivity in gastric cancer tissue and its relevance as a reliable prognostic marker for PD-L1/MET co-expression in gastric cancers.

PD-L1 positivity was observed in 31.2% gastric cancers, of which frequency was within the range (5.1%-65.0%) of previous studies in worldwide [9, 10, 16, 22, 23] and was more closely compatible with the PD-L1 rates (29.6%-50.8%) reported in the same Far East Asian population [8, 22-30]. Previous studies have reported conflicting data on correlations between PD-L1 expression in gastric cancers, suggesting both improved and poor prognoses. However, the majority of studies have shown a predominant correlation with poor survival [8, 22-24]. Some studies have reported favorable prognoses or no prognostic significance of PD-L1 expression [16, 26, 27, 30]. Nevertheless, we found that PD-L1 overexpression had a significant prognostic impact, resulting in decreased OS and DFS in patients with gastric cancer. A recently published meta-analysis is also consistent with the observation that PD-L1 overexpression can act as a significant biomarker for the poor prognosis of gastric cancers [29]. That study demonstrated that increased PD-L1 is associated with positive lymph node metastasis and poorer tumor stage, suggesting the involvement of tumor progression and metastatic potential [29]. Depth of invasion has been reported to be positively correlated with PD-L1 expression [8, 24]. Similarly, we found that advanced pT stage, as one of the adverse histopathological features, was a risk factor for PD-L1 overexpression in gastric cancers. PD-L1 positivity was also related to a lack of lymphatic invasion, which proved to be an independent risk factor for PD-L1 positivity. Kim et al. [28] also described a negative association between PD-L1 expression and lymphatic invasion with marginal significance (*P* = 0.067). The paradoxical correlation of PD-L1 expression with a lack of lymphatic invasion but poor prognosis may suggest that PD-L1-positive tumors may be related to other mechanisms coupled with other oncogenic pathways leading to metastatic potential.

PD-L1 expression has been reported to be driven by the MET/HGF oncogenic signaling pathway [12]. MET and HGF were expressed in 31.0% and 31.7% of gastric cancers, respectively, where approximately half of which were PD-L1 positive. PD-L1 expression was positively correlated with the overexpression of MET and HGF, which has been associated with adverse histological characteristics in many malignancies [13, 31-33]. However, only MET positivity, but not HGF, was an independent factor that was upregulated and affected PD-L1 overexpression as shown by a multivariate analysis. MET has also been shown to promote PD-L1 overexpression in esophageal squamous cell carcinoma and renal cell carcinoma [5, 7, 12]. In esophageal squamous cell carcinoma, high MET expression was the only independent factor affecting high PD-L1 expression [5]. MET expression is significantly correlated with PD-L1 positivity in clear cell renal cell carcinomas but not in papillary renal cell cancers [7]. However, those studies did not evaluate the prognostic value of PD-L1 and MET co-expression. Notably in our study, a significant positive correlation was observed between PD-L1/MET and prognosis; the worst outcome was observed in patients in the PD-L1+/MET+ subgroup when compared to those in the PD-L1-/MET- subgroup, which showed the best clinical outcome. PD-L1 and MET co-expression was shown to be an independent factor for poor prognosis that can be used to predict for decreased OS and DFS rates in patients with gastric cancers. Therefore, combined analyses of PD-L1 and MET expression may provide an accurate prediction for the prognosis of patients with gastric cancer. In particular, it would be valuable to help identify high-risk patients that are predicted to have the worst clinical outcome and in need of a more aggressive therapy after surgery, including a combination of chemotherapeutic agents or molecular-targeted therapy [34]. This may help guide the optimal selection of both PD-L1 and MET inhibitors for patient treatments. Since PD-L1/MET expression can be easily assessed in routinely processed tissue samples by immunohistochemistry, it could be employed as a promising prognostic tissue marker for patients with gastric cancer after resection. On the other hand, HGF expression seems to exhibit a limited clinical significance in PD-L1-positive gastric cancers.

Furthermore, PD-L1 overexpression in tumor cells inhibits T cell-mediated anti-tumor immunity via PD-1 on tumor infiltrating lymphocytes, which protects tumor cells from cytotoxic lysis and ultimately influences patients’ clinical outcomes [35]. The mechanism detailing how PD-L1 and MET affects survival has not been fully elucidated. Studies *in vitro* have shown that MET can modulate the survival of cancer cells through the regulation of PD-L1 [12, 14]. One plausible explanation may be that MET-induced PD-L1 expression is channeled through the Ras-PI3K pathway, which drives oncogenesis including the promotion of tumor cell invasiveness, angiogenesis, and the epithelial-to-mesenchymal transition [4, 6, 14, 31, 36]. Another possible explanation may be that HGF/MET signaling axis also plays important roles in the functional regulation of immune cells as well as affecting immunoregulatory properties [31]. MET-induced PD-L1 overexpression on cancer cells strengthens the immune escape of tumors through its interaction with PD-1 expressed on T cells or other immune cells [12]. Taken together, MET-induced PD-L1 ultimately leads to tumor progression and metastatic potential by an oncogenic pathway and by interrupting anti-tumor immunity. PD-L1+/MET+ group would be a subtype associated with oncogenic PD-L1 signaling in gastric cancers.

A recent phase Ib clinical trial (KEYNOTE-012) has shown that Pembrolizumab, an anti-PD-L1 monoclonal antibody, can result in an antitumor response rate as high as 22% in patients with PD-L1-positive advanced gastric cancer, thereby demonstrating the robust, durable responses of antibody therapy in patients with gastric cancer [9]. That study has shown PD-L1 expression in 65/162 (40%) patients with a trend towards improved outcomes with higher levels of PD-L1 expression [9], suggesting that a reliable determination of PD-L1 positivity is of great clinical importance when selecting treatments with PD-L1 inhibitors for patients with gastric cancer. PD-L1 expression was evaluated retrospectively on pre-treatment tumor tissue microarray sections by using a validated automated immunohistochemistry assay. Although the clinical trial categorized PD-L1 positivity as archival tumor PD-L1 staining in the stroma or in at least 1% of tumor cells [9], we used a cut-off value of >10% tumor cells as the most appropriate cutoff value among several cutoff points. A similar threshold was used to identify a cohort of gastric cancer cases in recent Korean cohort studies demonstrating statistical survival differences, thus providing an important external validation of the prior findings and supporting the use of this threshold [26, 28]. However, PD-L1 expression has been described as a favorable prognostic factor for Korean patients with gastric cancer [28], despite of application of same criteria, in contrast to our study. The possible explanation of contrasting results might be connected with postoperative chemotherapeutic agents in the patient cohort, which may be confounding factors associated with PD-L1. Expression of PD-L1 is upregulated by anti-cancer therapy including tyrosine kinase inhibitors [6]. Paclitaxel induces PD-L1 expression that is abolished by the MEK inhibitor U0126 [37]. Low concentration of cisplatin triggers the expression of PD-L1 through MAPK activation [6]. In the context, companion diagnostics for PD-L1 in gastric cancers is needed to reduce the confounding factors weakening the prognostic and predictive value of PD-L1. Nevertheless, that clinical trial study did not describe whether patients in the EBV-positive or MSI subgroups were associated with clinical benefits from the anti-PD-L1/PD-1 therapy, nor did any histopathological findings indicate the presence of high PD-L1 expression [9]. In our study, any subset of MSI, EBV, or mucin phenotypes did not represent a subgroup of patients expected to harbor PD-L1 overexpression. Rather, MET overexpression, high pT stage, and a lack of lymphatic invasion in gastric cancers represented histopathological features that were predictive of PD-L1 positivity. The overexpression of PD-L1 exhibited poor prognosis in EBV-negative, MSS, and intestinal mucin phenotype tumors, respectively. The frequencies of EBV and MSI-H in gastric cancers from our study were 6.6% and 9.4%, respectively, with the intestinal phenotype being the most common (39.8%), which was consistent with previous studies [16, 21, 38]. Few sufficient data are available concerning the prognostic relevance of PD-L1 expression according to EBV, MSI status, and mucin phenotype. Recently, Böger et al.[16] have shown that PD-L1 expression is prevalent in unclassified mucin phenotype, EBV-positive, and MSI-H gastric cancers. However, they did not investigate the prognostic correlation of PD-L1 expression according to MSI, EBV, or mucin phenotypes [16]. Derks et al. [15] also reported using a small series of 81 cases that PD-L1 expression is almost exclusively EBV-positive and MSI-H in gastric cancers. Furthermore, Cho et al. [26] have shown that PD-L1 expression is a favorable prognostic indicator of longer survival in patients with MSI-H gastric cancer. Additionally, 9p24 amplification is thought to lead to the overexpression of PD-L1 in EBV-associated gastric cancer [15]. However, a recent study has shown that PD-L1 expression was not restricted to gastric cancers with 9p24 amplification [15], which indicates that gastric cancers have multiple mechanisms to induce PD-L1 expression besides 9p24 amplification and suggests that PD-L1/PD-1-driven immune evasion may broadly play an important role in gastric cancers regardless of the prerequisite presence of EBV. Similar to our result showing a poor prognostic correlation of PD-L1 expression in EBV-negative gastric cancers, patients with diffuse large B-cell lymphoma expressing PD-L1 demonstrated inferior overall survival to those with EBV-negative tumors [39]. Although MSI-H gastric cancers are hyper-mutated, MSS gastric cancers also possess a high frequency of genetic alterations such as *CDH1*, *RHOA*, *HER2*, *EGFR*, *VEGFR*, *MET*, and *FGFR2,* all of which may directly or indirectly affect the MAPK and PI3K/Akt survival pathways [40, 41]. However, only a small number of EBV-positive (n = 26) or MSI-H (n = 37) tumors were included in our retrospective study; thus, our findings concerning the prognostic difference of PD-L1 according to EBV and MSI status should not be considered conclusive.

The limitations of our study include the retrospective procurement of archival samples. Nevertheless, our results raise some interesting points. The PD-L1 expression had clear poor prognostic significances in patients with gastric cancer, indicating the potential of PD-L1 as a prognostic tissue marker. The subgroup analysis of PD-L1/MET co-expression could suggest the detailed prognostic prediction after surgery. This may help to guide the selection of treatments for high-risk patients in need of more intensive therapy and the possibility of anti-PD-L1 targeted therapy in this subset of gastric cancers.

## MATERIALS AND METHODS

### Patients and histological evaluation

Gastric cancer tissue samples were retrospectively collected from 394 patients (stages IB to IVa) who underwent gastrectomy with extensive node dissection (D2) consecutively from 2003 to 2011 at Hallym University Sacred-Heart Hospital, Korea. Only those patients who were diagnosed with primary gastric cancers and that were not treated with chemotherapy and targeted drug therapy at the time of the surgery, and whose formalin-fixed, paraffin-embedded (FFPE) tumor tissue blocks were available for analysis were included in this study. Medical records of each patient were reviewed for demographic information, radiological data, treatment details, tumor recurrence, or survival status. All of the slides stained with hematoxylin and eosin were reviewed by two pathologists (MJK and JYC) for diagnostic confirmation, the re-evaluation for histopathological characteristics (Lauren type, histologic type, histologic grade, depth of invasion, lymphatic invasion, venous invasion, and perineural invasion), and the selection of a representative section for subsequent immunohistochemical and molecular studies. The diagnostic criteria and tumor differentiation metrics used were those indicated by the World Health Organization classification of tumors of the digestive system. Tumor stage was defined according to the TNM classification of malignant tumors as described in the 7^th^ edition of the American Joint Committee on Cancer (AJCC) cancer staging manual. This study was approved by the Institutional Review Board of Hallym University Sacred-Heart Hospital (2017-I037).

### Tissue microarray and immunohistochemistry

Tissue microarrays were constructed for each FFPE block as described previously [32]. After reviewing the tumor sections, three tissue cores (3 mm in diameter) from the invasive front, lateral side, and the luminal surface of the representative tumor block for each primary carcinoma were obtained separately from each case and arranged in the new recipient tissue microarray blocks using a trephine apparatus (Quick-Ray™; Unitma, Seoul, South Korea). Immunohistochemical staining was performed on 4-μm-thick sections using BenchMark XT automated stainer (Ventana Medical System, Tucson, AZ, USA) according to the manufacturer’s instructions. Primary antibodies used were PD-L1 (rabbit anti-human PD-L1 monoclonal, 1:25, clone SP142; Ventana Medical System), MET (rabbit polyclonal anti-c-Met, pre-diluted; Ventana Medical System), HGF (1:50; Epitomics, Burlingame, CA, USA), MUC2 (Ccp58, 1:100, Novocastra, Newcastle, UK), MUC5AC (CLH2, 1:100, Novocastra), MUC6 (CLH5, 1:50, Novocastra), and CDX-2 (1:50 dilution; Biogenex, San Ramon, CA, USA) for 40 min at 37°C, then a secondary antibody of Universal HRP Multimer (Ventana Medical System) was used for 8 min at 37°C. The tissue sections were then incubated with the chromogen diaminobenzidine (ultraView Universal DAB Kit, Ventana Medical System) and were counterstained with hematoxylin.

**Figure 3:**
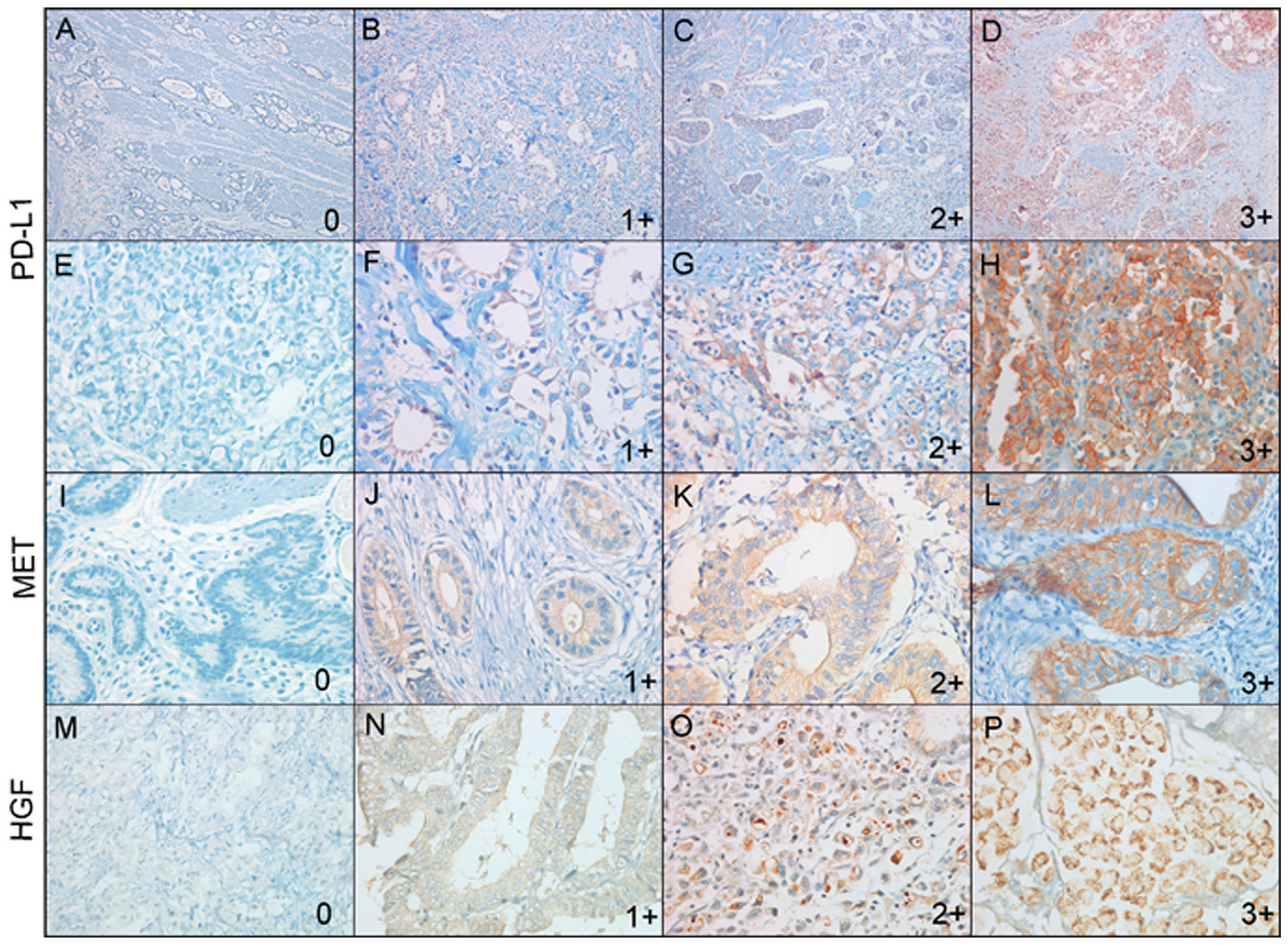
Representative immunohistochemical images of PD-L1, MET, and HGF expressions in gastric carcinomas PD-L1 expression was scored as 0 **(A, E)**, 1+ **(B, F)**, 2+ **(C, G)**, and 3+ **(D, H)**. Low power view (x100) (A)-(D) and high power view (x400) (E-H) of PD-L1 expression. MET expression was scored as 0 (E), 1+ (F), 2+ (G), and 3+ (H). HGF expression was scored as 0 **(I)**, 1+ **(J)**, 2+ **(K)**, and 3+ **(L).**

PD-L1 was evaluated based on the intensity and proportion of membranous staining and/or cytoplasmic staining in tumor cells. PD-L1 was scored as 0 (no staining or any staining less than 10% of cells), 1+ (weak staining in more than 10% of the tumor cells), 2+ (moderate staining in more than 10% of the tumor cells), or 3+ (strong staining in more than 10% of the tumor cells) [26, 28] (Figure 3A-3H). Cases with scores of 1+, 2+, or 3+ were considered to be PD-L1-positive [26, 28]. Because most prognostic factors are considered as dichotomized and discontinuous variables, the best appropriate cutoff point among several candidate cutoff points was selected to give the optimal separation between low risk and high risk for OS.

Staining score of MET and HGF expression using modified criteria used in clinical trials involving the MET inhibitor was defined as the following: 0, absence of staining or staining intensity in <50% tumor cells; 1+, weak-to-moderate staining intensity in >50% tumor cells; 2+, moderate to strong intensity staining in >50% of tumor cells; 3+, strong staining intensity in >50% tumor cells based on membranous and/or cytoplasmic staining (Figure 3I-3L for MET and Figure 3M-3P for HGF) [33, 42]. Cases with a score of 2+ or 3+ were classified as MET- or HGF-positive, and those with a score of 0 or 1+ were classified as MET- or HGF-negative, respectively [43].

**Figure 4:**
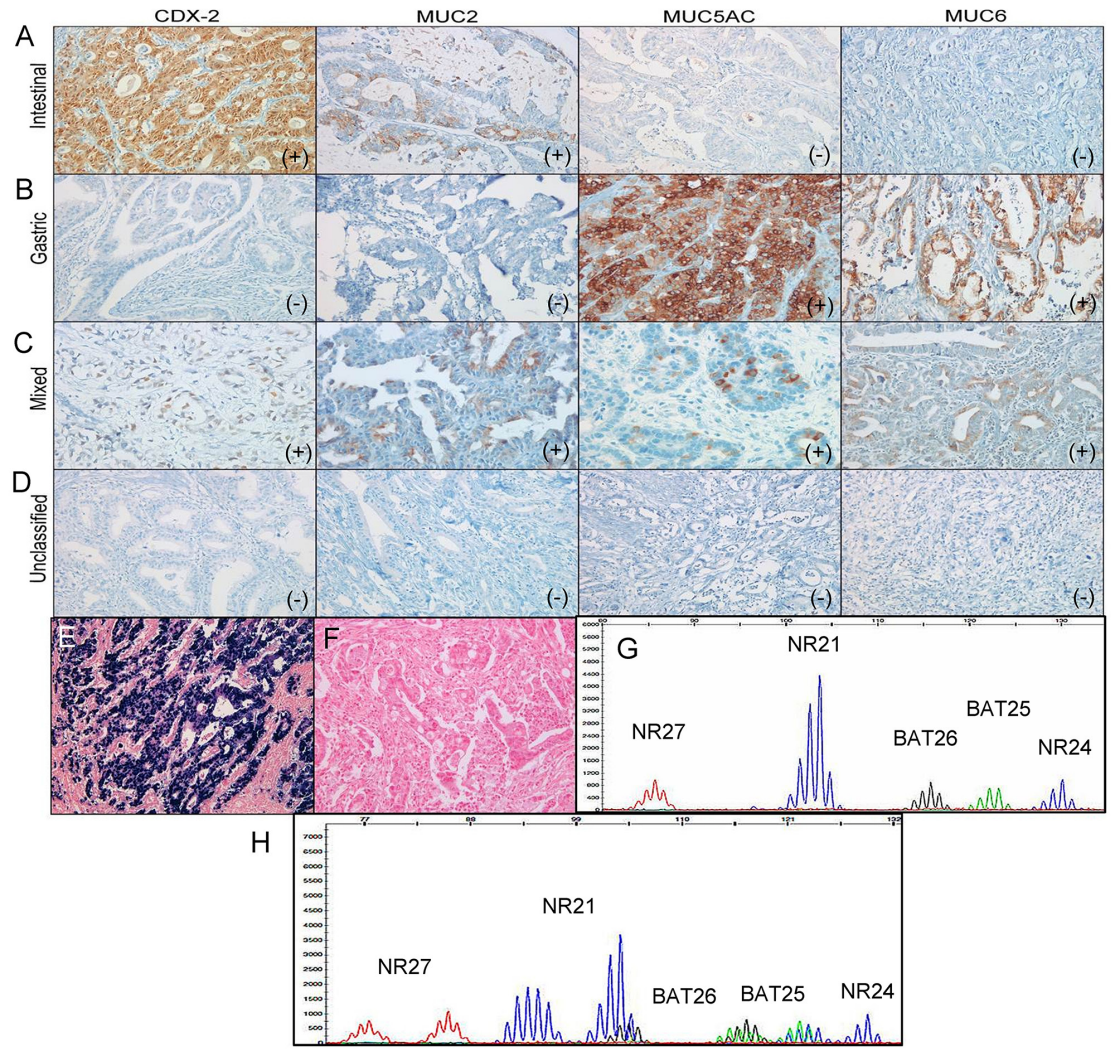
Representative images of intestinal **(A)**, gastric **(B)**, mixed **(C)**, and unclassified **(D)** mucin phenotypes based on mucin marker expressions of CDX-2, MUC2, MUC5AC, and MUC6 in gastric carcinomas. Representative images of EBV-positive **(E)** and EBV-negative **(F)** gastric carcinomas. **(G)** Microsatellite stable status. **(H)** Microsatellite instability-high status.

CDX-2 expression was regarded as positive if nuclear staining was observed in ≥5% of the tumor specimens; otherwise, it was classified as negative according to previous reports [44, 45]. For MUC2, MUC5AC, and MUC6, positive staining was defined as distinct membranous and cytoplasmic staining in more than 5% of the tumor cells [44, 45]. Four mucin phenotype subgroups were classified according to their immunostaining pattern for CDX-2, MUC2, MUC5AC, and MUC6. An intestinal phenotype was defined as a tumor with positive immunostaining with CDX-2 and/or MUC2 (Figure 4A). Gastric phenotype cancer was defined as a tumor with positive immunostaining with MUC5AC and/or of MUC6, but negative for CDX-2 and/or MUC2 (Figure 4B). A mixed phenotype was defined as a tumor with characteristics of both intestinal and gastric phenotypes (Figure 4C). Unclassified phenotype was classified as a tumor without positive immunostaining consistent with either gastric or intestinal phenotypes (Figure 4D). Two pathologists independently scored all samples, and cases with discrepant scores were re-evaluated to achieve a consensus score.

### EBV-encoded RNA *in situ* hybridization

*In situ* hybridization for EBV-encoded RNA was also performed on the BenchMark XT autostainer using fluorescein-conjugated oligonucleotide probes (EBER Probe, Ventana Medical System). In brief, 3-μm-thick sections from the tissue microarray blocks were deparaffinized and rehydrated, and then the sections were digested with a proteolytic enzyme (proteinase K at 37°C for 25 min). Thereafter, the slides were incubated with the probe at 55°C for 25 min and washed with a stringent solution. The slides were labeled with an anti-alkaline phosphatase-conjugated antibody to fluorescein. A chromogen (5-bromo-4-chloro-3-indolylphosphate and nitroblue tetrazolium) was then added and counterstained with Mayer’s hematoxylin. Only cases with a strong signal within almost all tumor cell nuclei were considered positive (Figure 4E-4F).

### Microsatellite instability analysis

Tumor DNA was extracted from 10-μm-thick sections of FFPE tumor tissue blocks from individual patients. The MSI test was performed in all patients using multiplex PCR with five quasi-monomorphic markers (BAT25, BAT26, NR21, NR24, and NR27), as previously described [20, 26]. In brief, each sense primer was end-labeled with one of the following fiuorescent markers: FAM, HEX, or NED. Pentaplex PCR was performed with an initial 15 min denaturation at 94°C, followed by 35 cycles at 94°C for 30 s, 55°C for 30 s, and 72°C for 30 s, with a final extension at 72°C for 10 min. Amplicons were analyzed on an ABI Prism 3130 Genetic Analyzer (Applied Biosystems). Allelic sizes were estimated using Genemapper® 4.1 software (Applied Biosystems). Tumors with no allelic size variations or allelic size variations in fewer than two of the microsatellites were classified as MSS (Figure 4G), whereas those with allelic size variations in two or more of the microsatellite markers were considered MSI-H (Figure 4H). In cases with equivocal cases, the additional immunohistochemical stains for MLH1, MSH2, MSH6, and PMS2 were performed.

The MSI evaluation was performed using multiplex PCR comprising five quasimonomorphic mononucleotide repeat markers (NR27, NR21, NR24, BAT25, and BAT26) [20]. Each sense primer was end-labeled with one of the fiuorescent markers, FAM, HEX, or NED. Pentaplex PCR was performed with an initial 5-min denaturation step at 948C, followed by 35 cycles at 94C8 for 30 sec, 558C for 30 sec, and 728C for 30 sec with a final extension at 728C for 10 min. Amplified PCR products were run on an Applied Biosystems PRISM 3130 automated genetic analyzer. Allelic sizes were estimated using Genescan 2.1 software (Applied Biosystems, Foster City, LA). Samples with no allelic size variations in any of the microsatellites were classified as microsatellite stable (MSS). Tumors with allelic size variations in fewer than three of the microsatellites were classified as MSI-low, whereas those with allelic size variations in three or more of the microsatellites were considered MSI-high

### Statistical analysis

Categorical variables were compared using Pearson’s chi-squared test or Fisher’s exact test, and continuous variables, which are presented as means ± SD, using the t-test. Factors found to be significant (*P* <0.05) in univariate analysis were included in subsequent multivariate logistic regression analysis to identify independent variables associated with PD-L1 and MET expression. Survival analyses were performed using the Kaplan-Meier method and were compared using a log-rank test. OS was defined as the interval from the first day of surgery until death or the end of the follow-up period. DFS was defined as the interval from the first day of surgery until tumor progression, death, or end of follow-up period. OS and DFS were analyzed until February 2015. Univariate and multivariate analyses using the Cox proportional hazard regression model were applied to determine the HR and 95% CI for specific variables with respect to OS and DFS. SPSS version 18 (SPSS Inc., Chicago, IL, USA) was used for all statistical analyses. A *P* value <0.05 was considered statistically significant.
